# Streamlining Patient Management of Suspected COVID-19 Patients in the Emergency Department: Incorporation of Pulmonary CT Angiography into the Triaging Algorithm

**DOI:** 10.3390/diagnostics12051183

**Published:** 2022-05-09

**Authors:** Benedikt M. Schaarschmidt, David Fistera, Yan Li, Margarete Konik, Johannes Haubold, Johannes Grueneisen, Oliver Witzke, Michael Forsting, Carola Holzner, Lale Umutlu

**Affiliations:** 1 Institute of Diagnostic and Interventional Radiology and Neuroradiology, University Hospital Essen, 45147 Essen, Germany; yan.li@uk-essen.de (Y.L.); johannes.haubold@uk-essen.de (J.H.); johannes.grueneisen@uk-essen.de (J.G.); michael.forsting@uk-essen.de (M.F.); lale.umutlu@uk-essen.de (L.U.); 2 Center for Emergency Medicine, Universitätsmedizin Essen, 45147 Essen, Germany; david.fistera@uk-essen.de (D.F.); carola.holzner@uk-essen.de (C.H.); 3 Department of Infectious Diseases, West German Centre of Infectious Diseases, Universitätsmedizin Essen, 45147 Essen, Germany; margarethe.konik@uk-essen.de (M.K.); oliver.witzke@uk-essen.de (O.W.)

**Keywords:** computed tomography, CT, angiography, COVID-19, pneumonia

## Abstract

Purpose: To evaluate the use of pulmonary computed tomography (CT) angiography during initial admission at an emergency department (ED), to identify COVID-19 patients with accompanying pulmonary embolism (PE) and its impact on clinical management. Methods: We performed a retrospective analysis of COVID-19 patients that underwent pulmonary CT angiography at the ED. CT scans were evaluated for the presence and extent of PE and for imaging changes suspicious of COVID-19. Patients were subdivided into two groups: (1) Group A consisted of patients with proven COVID-19 based on real-time polymerase chain reaction (RT-PCR), and (2) Group B of patients suspected for COVID-19, comprising patients positive on RT-PCR and/or COVID-19-suspicious CT findings. To assess the differences between patients with and without pulmonary embolism, Fisher’s exact test was used. Results: A total of 308 patients were admitted to the ED for diagnostic work-up of dyspnea and suspected COVID-19, and 95 patients underwent pulmonary CT angiography. PE was detected in 13.6% (3/22) of patients in Group A and 20.7% (6/29) in Group B. No significant differences were observed between patients with and without PE concerning hospitalization (Group B: 100% (6/6) vs. 91.3% (21/23)), the necessity of oxygen therapy (Group B: 66% (4/6) vs. 43.5% (10/23)), and death (Group B: 33% (2/6) vs. 4.3% (1/23) *p* > 0.05, respectively). Conclusions: In 20.7% of COVID-19 patients, PE was detected upon admission to the ED. Although the incorporation of early pulmonary CT angiography in patients suspicious of COVID-19 may be beneficial to identify concomitant PE, further studies are necessary to corroborate these findings.

## 1. Introduction

The ongoing COVID-19 pandemic poses unforeseen problems to healthcare systems around the world. At the beginning of the pandemic, there was a lack of protective clothing or accessories, such as face shields or masks, making it difficult to protect medical personnel and uninfected patients, even in highly developed countries. At the moment, the high incidence rates pose new challenges for triage algorithms. However, the detection of viral nucleic acid by real-time polymerase chain reaction (RT-PCR) in patients with suspected SARS-CoV-2 infection is still time-consuming. Therefore, workflows need to be further optimized that allow for a rapid and accurate diagnosis to identify patients at risk of severe disease progression, minimize contact between infected patients and medical staff to reduce the risk of infection, and reduce the use of medical resources [1,2]. 

Initial reports from China indicated that distinctive imaging patterns on non-contrast-enhanced computed tomography (CT) examinations can be used to identify patients suffering from COVID-19 [3,4,5]. However, recent recommendations stress that these CT findings should be considered unspecific, especially early in the outbreak in Western countries. Therefore, a number of guidelines do not recommend CT as an alternative to RT-PCR to identify COVID-19 patients [6,7,8]. Still, imaging might play a pivotal role in assessing disease extent over the course of the disease [9,10,11]. Furthermore, recent reports on patients with COVID-19 and concomitant pulmonary embolism (PE) indicate the association of this disease with coagulation disorders and stress the need for further diagnostic testing [12,13,14,15,16,17]. These initial reports were confirmed by a recent study by Ward et al. that found an association between COVID-19 and a higher 90-day risk of venous thrombosis compared with influenza [18]. 

Therefore, the current literature emphasizes the urgency to confirm or exclude the diagnosis of PE in these patients as early as possible, preferably during the first admission to the emergency department (ED). Although several tests are available to identify or at least exclude PE, they are not without shortcomings. Laboratory D-dimer testing has a low positive predictive value and therefore can only be used to exclude the presence of thromboembolic events [19]. However, elevated D-dimer levels are frequently found in COVID-19 patients during and even three months after recovery from the disease and are therefore of limited clinical use in patients with suspected SARS-CoV-2 infection [20,21,22]. Lung perfusion scintigraphy increases the exposure between patients and medical staff by necessitating additional chest X-ray or chest CT examinations to exclude lung opacities [23]. Consequently, pulmonary CT angiography may be a highly valuable option for quick patient assessment of PE and the extent of parenchymal changes in potential COVID-19 patients [24]. Therefore, the aim of the present study was to evaluate the use of pulmonary CT angiography in the initial clinical pathway to detect the presence of PE in COVID-19 patients at initial admission to the ED in the early phase of the pandemic. 

## 2. Materials and Methods

### 2.1. Clinical Pathway at the Emergency Department

To improve the clinical workflow, reduce the contact between medical staff and potentially infectious patients, and minimize the number of examinations, a streamlined diagnostic concept for patients with respiratory problems was established in our ED in March 2020 to address the needs of the ongoing COVID-19 pandemic. After the admission of each patient to the ED, a Manchester triage that included the acquisition of basic vital parameters (blood pressure, heart rate, breathing frequency, oxygen saturation, temperature) was performed as a short clinical assessment by an experienced physician trained in emergency medicine. Then, a second physician performed a second, more profound, focused clinical examination. In patients with moderate to severe symptoms, point-of-care testing and a nasopharyngeal swap supplemented this preliminary assessment for SARS-CoV-2 RT-PCR. 

In patients with suspected lower respiratory tract involvement or a non-infectious cause defined by dyspnea, low oxygen saturation, increased breathing frequency, coughing or clinical suspicion for other causes due to clinical tests (e.g., positive Wells score), the following tests were performed: ECG, blood gas analysis, and advanced laboratory testing, including leucocyte and lymphocyte count, C-reactive protein (CRP), procalcitonine (PCT), D-dimer testing and pulmonary CT angiography. Based on the results, patients were either discharged for outpatient treatment or transferred to a regular ward, an intermediate or an intensive care unit.

### 2.2. Patients

**Figure 1:**
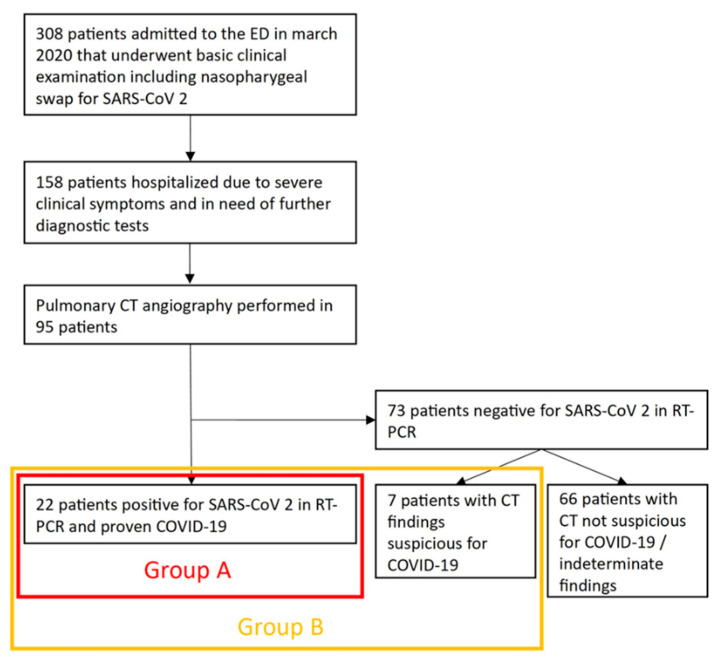
Flowchart of patients included in the study.

Over the course of one month in spring 2020, 308 patients were treated in our ED for dyspnea with a suspected SARS-CoV-2 infection according to the new COVID-19-specific workflow (Figure 1). A total of 95 patients (female: 34.7% (33/95); male: 65.3% (62/95); mean age: 66.15 ± 17.06 years), who underwent pulmonary CT angiography in the analyzed timeframe at our ED, were included in this retrospective analysis. This study was approved by the institutional review board of the University of Duisburg-Essen (application number: 20-9231-BO, approval date: 7 April 2020). Due to the retrospective nature of this study, informed consent was waived for all patients.

### 2.3. Acquisition of Pulmonary CT Angiography

CT scans were acquired in the supine position on a Siemens Somatom Force CT (Siemens Healthineers, Erlangen, Germany). A total of 60 mL of contrast agent (Ultravist 300, Bayer, Berlin, Germany) was injected via a venous 18 G line. Bolus triggering was performed by placing a region of interest into the main pulmonary artery with a threshold of 90 Houndsfield units. CT images were acquired 3 s after the threshold was reached in flash mode (Collimation: 192 × 0.6 mm, pitch: 1.55, rotation time: 0.25 s). Care dose (reference setting: 174 mAs) and CarekV (reference setting: 80 kV) were used to reduce the patient dose. Images were reconstructed using a lung (Bv59) and a soft tissue kernel (Bv36) in 1 mm and 5 mm slice thicknesses. Advanced modeled iterative reconstruction (ADMIRE, level 3) was used to improve the image quality.

### 2.4. Data Collection and Image Interpretation

Clinical data were collected from the clinical reports, including preexisting illnesses, clinical symptoms at admission (fever, cough, fatigue, sputum production, shortness of breath, myalgia or arthralgia, dysgeusia, headache, sore throat, chills, nausea/vomiting/diarrhea, discomfort in the chest), basic vital parameters at admission (blood pressure, heart rate, breathing frequency, oxygen saturation, temperature), results from laboratory testing at admission (leucocyte and lymphocyte count, C-reactive protein (CRP), procalcitonine, D-dimer), and the results from RT-PCRs. Application of oxygen therapy, as well as the date of discharge and, in case of hospitalization, the type of ward, was recorded. 

CT examinations were reviewed by two experienced board-certified radiologists for the presence of PE and infectious changes of lung parenchyma. The extent of PE was assessed according to the Qanadli Score [25]. Here, the presence of thrombotic material in each of the segmental arteries or their proximal arterial level is rated with one point, and in the case of total vessel occlusion, a weighting factor of 2 can be applied, resulting in a maximum score of 40. Infectious changes in the lungs were scored according to the CO-RADS criteria [26,27] (Table 1).

The following CT findings were considered typical of COVID-19 infection: peripheral distribution of multifocal ground-glass opacities, consolidations and the absence of pleural effusion [28,29]. If a Co-RADS score greater than 4 was assigned by either investigator, the CT severity score was also assessed [9]. Here, the extent of pulmonary opacifications was rated with 1–4 points for each lobe, resulting in a maximum score of 20. Disagreements between the two readers regarding the presence of PE were resolved by consensus reading.

### 2.5. Reference Standard

Although RT-PCR is still considered the gold standard for the diagnosis of SARS-CoV-2 infection due to its high specificity, a recent study by Ai et al. [4] indicated that this diagnostic test is not sensitive enough to detect early disease. Based on the reported high specificity of lung CT for the diagnosis of COVID-19 [29], we decided to define two reference standards as follows. The results for both reference standards are reported separately in the results section.

1. Group A: Patients with positive RT-PCR results for SARS-CoV-2 infections.
2. Group B: Patients with positive RT-PCR results for SARS-CoV-2 infections and/or CT findings reported as suspicious for COVID-19 by at least one radiologist.

### 2.6. Statistical Analysis

We performed an exploratory data analysis using IBM SPSS Statistics 26 (IBM, Armonk, NY, USA). The values are given either as the frequency (%) or as mean values, including standard deviation. For Co-RADS, Cohen’s kappa, as well as the Spearman’s rank correlation coefficient, were calculated to analyze the inter-reader agreement. To assess differences between the two groups, we used categorical data Fisher’s exact test. As we could not assume a normal distribution for continuous values due to the low patient count, a Mann–Whitney U test was used. A *p*-value < 0.05 was considered statistically significant. 

## 3. Results

The clinical characteristics of all patients are displayed in Table 2. According to the results from RT-PCR, 23.2% of patients were positive for SARS-CoV-2 (Group A, 22/95).

In seven additional patients, at least one reader considered the CT findings as suspicious of COVID-19, thus, 30.5% of all patients were considered potentially COVID-19 positive (Group B, 29/95).

### 3.1. CT Imaging

PE on CT was detected in 13.7% of all patients (13/95) with a mean Qanadli score of 6.5 ± 5.1 (out of a maximum of 40 points, indicating complete occlusion of all pulmonary arteries). In Group B, PE was found in 20.7% (6/29) with a mean Qanadli score of 7.8 ± 6.7. In patients with proven COVID-19 (Group A), PE was detected in 13.6% (3/22) with a mean Qanadli score of 5.8 ± 2.6.

CT findings were classified as suspicious of COVID-19 (Co-RADS 4) in seven patients by reader 1 and in 12 patients by reader 2. Both readers regarded the CT changes as typical for COVID-19 in six patients (Co-RADS 5). A total of seven patients had a positive RT-PCR for SARS-CoV-2 prior to CT imaging and were therefore classified as Co-RADS 6 by both readers. CT findings were considered as non-typical for COVID-19 in 66 patients by reader 1 (Co-RADS 1: *n* = 28; Co-RADS 2: *n* = 38) and in 53 patients by reader 2 (Co-RADS 1: *n* = 24; Co-RADS 2: *n* = 29). CT findings were considered indeterminate in 9 patients by reader 1 and 17 patients by reader 2 (Co-RADS 3). Detailed results from the CO-RADS analysis are presented in Table 3. Spearman’s rank correlation coefficient between both readers was very good for the Co-RADS analysis (rs = 0.88, *p* < 0.001) and good for Cohen’s kappa between both readers (κ = 0.68, *p* < 0.001).

No significant differences could be observed for the COVID-19 CT severity score between patients with and without PE, nor in patients with proven COVID-19 (Group A: with PE: 4.8 ± 4.3; without PE: 6.7 ± 3.5; *p* = 0.523) nor patients with suspected COVID-19 (Group B: with PE: 6.1 ± 5.4; without PE: 6.4 ± 3.8; *p* = 0.655).

### 3.2. Clinical and Laboratory Parameters

COVID-19 patients with PE tended to have higher D-dimer values (Group A: 13.5 ± 10.9; Group B: 13.2 ± 14.0) compared to patients without PE (Group A: 5.0 ± 8.9; Group B: 4.6 ± 8.3). However, no significant differences in D-dimer values were detected using the Mann–Whitney U test (Group A: *p* = 0.132; Group B: *p* = 0.112). 

Clinical outcome parameters yielded similar results in both groups for patients with and without PE. No significant differences were observed concerning hospitalization (Group A: patients without PE: 94.7% (18/19), patients with PE: 100% (3/3), *p* = 1.0; Group B: patients without PE: 91.3% (21/23), patients with PE: 100% (6/6), *p* = 1.0); necessity of intermediate and/or intensive care (Group A: patients without PE: 5.2% (1/19), patients with PE: 0% (0/3), *p* = 1.0; Group B: patients without PE: 4.3% (1/23), patients with PE: 0% (6/6), *p* = 1.0); necessity of oxygen therapy and/or mechanical ventilation (Group A: patients without PE: 36.8% (17/19), patients with PE: 66% (2/3), *p* = 0.544; Group B: patients without PE: 43.5% (10/23), patients with PE: 66% (4/6), *p* = 0.390); length of hospital stay (Group A: patients without PE: 10.0 ± 8.1, patients with PE: 9 ± 3, *p* = 0.787; Group B: patients without PE: 9.5 ± 7.6, patients with PE: 5.5 ± 4.3, *p* = 0.302); or death (Group A: patients without PE: 5.2% (1/19), patients with PE: 33% (1/3), *p* = 0.260; Group B: patients without PE: 4.3% (1/23), patients with PE: 33% (2/6), *p* = 0.100, see Table 4).

## 4. Discussion

Since the beginning of this global pandemic, CT imaging has been considered an important diagnostic tool in the detection of COVID-19, enabling early and better identification of lung involvement [4,11,28]. Nevertheless, up to its current status, RT-PCR is still considered the gold standard for the diagnosis of SARS-CoV-2 infections due to its high specificity, as recently demonstrated by Ai et al. [4]. Hence, despite the promising results of the first studies, recent recommendations opposed the initial idea of using non-contrast-enhanced CT as a screening tool to identify patients suffering from a SARS-CoV-2 infection [3,4,5]. Especially in Western countries with a low pretest probability for COVID-19, most radiological societies do not yet recommend CT as a tool to screen or identify COVID-19-positive patients [7,8]. Nonetheless, the role of CT imaging in COVID-19 remains to be pivotal: first, to assess disease extent, which might be an important prognostic factor [9], and second, to identify and/or exclude other causes of dyspnea. Therefore, we aimed to evaluate a new clinical pathway in our ED that included pulmonary CT angiography to detect PE in COVID-19 patients at initial admission.

In an effort to streamline clinical pathways in the ED during this pandemic, an important step was to include CT for suspected COVID-19 in the early steps of the patient triaging algorithm. One of the most thoroughly discussed issues was the administration of contrast agents. Most initial studies underlining the importance of CT imaging in COVID-19 recommended the application of non-contrast-enhanced CT imaging [3,4,5]. While this enables the identification of potential lung parenchymal involvement, other causes of dyspnea, such as PE, are debarred from detection. The importance of contrast application and thus the exclusion of PE is emphasized by the fact that the current literature indicates coagulopathic disorders and a high probability of elevated D-dimer values in patients suffering from COVID-19 [18,21,30,31,32].

To ensure efficient diagnostics while minimizing the exposure of medical staff and other patients to potential COVID-19 patients, pulmonary CT angiography was included early in the patient triaging algorithm in our emergency department. The decision to include contrast application is also supported by a recent study published by Poissy et al. [33]. They raised awareness of an increased prevalence of PE in COVID-19 patients, which was evident in their results of an observational study among the first 107 consecutive confirmed COVID-19 patients admitted to the intensive care unit ICU for pneumonia from mid-February to the end of March 2020. While the overall number of PE in COVID-19 patients is comparable to our results (20.6% vs. 20.7%, respectively), the most pivotal difference between the two studies lies in the time of PE detection. Poissy et al. performed the pulmonary CT angiography scans for exclusion/confirmation of PE at an average of 6 days after ICU admission. On the contrary, in our study, pulmonary CT angiography was performed on the initial day of admission into the ED, suggesting that the development of PE may occur much earlier in the course of the disease than anticipated according to the results of Poissy et al. [33]. In addition, our findings are consistent with other recent studies on PE and COVID-19, revealing a comparably high incidence of PE in COVID-19 patients (>20%) compared to patients in general EDs (3–10%) [16,33,34]. These findings have also been confirmed by autopsy studies where, in a recent meta-analysis, Zuin et al. reported acute PE in 30% of COVID-19 patients. Moreover, PE was the underlying cause of death in almost 20% of cases [35]. Therefore, the proposed changes in the diagnostic workflow may be useful to ensure effective thromboprophylaxis and early PE treatment in COVID-19 patients [32,36].

Considering D-dimer levels and their association with the prevalence of PE, our results highlight an anticipated association between elevated D-dimer values and the increased likelihood of PE. However, in contrast to the results of Léonard-Lorant et al., our results on laboratory and clinical parameters did not show significant correlations with clinical outcomes [16]. This could be due to the early recognition of PE and the corresponding change in patient management; however, further studies with larger patient cohorts are needed in this regard.

Our study has limitations. First, we performed a retrospective analysis. Therefore, no benefit of this new algorithm for patient triage in terms of outcome parameters such as survival can be derived from the current data. Here, a prospective randomized trial is necessary to evaluate its potential advantages. Second, the study data do not comprise patients suffering from new virus variants, such as delta or omicron. Therefore, continued data analysis on this topic would be desirable.

## 5. Conclusions

Overall, putting our result into perspective to the current literature in these early stages of the pandemic in Western countries, our results go in line with the current literature in underlining the higher prevalence of pulmonary embolisms in COVID-19 patients, while emphasizing the potential of pulmonary CT angiography as an early detection tool in a streamlined COVID-19 patient triaging algorithm. However, further studies are necessary to elucidate the benefit of this new patient triaging algorithm concerning overall survival.

## Figures and Tables

**Table 1:** Co-RADS criteria [26,27].

Score	CT Findings	Level of Suspicion for COVID-19 Infection
Co-RADS 1	Normal CT or definitely non-infectious CT findings	
Co-RADS 2	CT findings consistent with infections other than COVID-19	low
Co-RADS 3	Unclear whether COVID-19 is present	indeterminate
Co-RADS 4	Abnormalities suspicious of COVID-19	high
Co-RADS 5	Typical COVID-19 findings	very high
Co-RADS 6		proven SARS-CoV-2 infection by RT-PCR

**Table 2:** Clinical characteristics of patients treated at the ED, including subgroup analysis of proven and probable COVID-19 patients.

	All Patients (*n* = 95)	Group A (*n* = 22)	Group B (*n* = 29)
**Sex**			
female	34.7% (33/95)	22.7% (5/22)	31.0% (9/29)
male	65.3% (62/95)	77.3% (17/22)	69.0% (20/29)
			
**Age (years)**	66.2 ± 17.1	71.1 ± 12.7	70.6 ± 13.7
			
**Nicotine abuse**			
never	30.5% (29/95)	36.4% (8/22)	27.6% (8/29)
active smoker	7.4% (7/95)	0% (0/22)	3.4% (1/29)
former smoker	17.9% (17/95)	22.7% (5/22)	20.7% (6/29)
unknown	44.2% ((42/95)	40.9% (9/22)	48.3% (14/29)
			
**Preexisting illnesses**			
cardiovascular	64.2% (61/95)	54.5% (12/22)	48.3% (14/29)
nephrological	11.6% (11/95)	0% (0/22)	0% (0/29)
pulmonary	18.9% (18/95)	22.7% (5/22)	27.6% (8/29)
gastrointestinal	21.1% (20/95)	18.2% (4/22)	17.2% (5/29)
endocrinological	22.1% (21/95)	31.8% (7/22)	31.0% (9/29)
rheumatological	3.2% (3/95)	9.1% (2/22)	6.9% (2/29)
neurological	27.4% (26/95)	27.3% (6/22)	34.5% (10/29)
oncological	22.1% (21/95)	9.1% (2/22)	13.8% (4/29)
immunosupression	10.5% (10/95)	13.6% (3/22)	10.3% (3/29)
transplantation	4.2% (4/95)	0% (0/29)	0% (0/29)
			
**Clinical symptoms**			
fever	40.0% (38/95)	40.9% (9/22)	37.9% (11/29)
cough	35.8% (34/95)	50.0% (10/20)	41.4% (12/29)
fatique	40.9% (38/93)	4.5% (1/22)	48,1% (13/27)
sputum production	7.4% (7/94)	0% (0/21)	3.6% (1/28)
shortness of breath	54.3% (51/94)	38.1% (8/21)	46.4% (13/28)
myalgia or arthralgia	15.1% (14/93)	18.2% (4/22)	3.8% (4/29)
dysgeusia	6.5% (6/92)	14.3% (3/21)	14.3% (4/28)
headache	11.0% (10/91)	13.6% (3/22)	10.3% (3/29)
sore throat	7.6% (7/92)	9.5% (2/21)	7.1% (2/28)
chills	9.7% (9/93)	4.5% (1/22)	3.4% (1/29)
nausea/vomiting/diarrhea	23.7% (22/93)	13.6% (3/22)	17.9% (5/28)
discomfort in the chest	13.7% (13/95)	4.5% (1/22)	3.4% (1/29)
**Basic vital parameters**			
blood pressure (mmHg)			
systolic	131.1 ± 22.7 (*n* = 95)	139.3 ± 21.5 (*n* = 22)	135.2 ± 22.1 (*n* = 29)
diastolic	127.0 ± 82.0 (*n* = 95)	86.3 ± 15.9 (*n* = 22)	86.7 ± 16.3 (*n* = 29)
heart rate	94 ± 21.5 (*n* = 95)	92.0 ± 17.3 (*n* = 22)	94.5 ± 18.1 (*n* = 29)
breathing frequency	21 ± 7.0 (*n* = 91)	20.4 ± 5.5 (*n* = 22)	20.5 ± 5.5 (*n* = 29)
oxygen saturation	93.4 ± 8.6 (*n* = 93)	94.1 ± 5.1 (*n* = 22)	94 ± 5.3 (*n* = 29)
temperature (°C)	37.0 ± 1.2 (*n* = 82)	36.7 ± 1.1 (*n* = 22)	36.6 ± 1.2 (*n* = 27)
			
**Laboratory tests**			
CRP (<0.5 mg/dL)	8.7 ± 9.6 (*n* = 95)	10.0 ± 5.3 (*n* = 22)	10.5 ± 7.9 (*n* = 29)
PCT (0–0.5 ng/mL)	1.5 ± 6.5 (*n* = 87)	0.1 ± 0.1 (*n* = 22)	0.4 ± 1.3 (*n* = 29)
D-dimer (<0.5 mg/dL)	5.6 ± 9.0 (*n* = 90)	6.1 ± 9.4 (*n* = 22)	6.5 ± 10.2 (*n* = 28)
Leucocytes (3.6–9.2 nL)	9.7 ± 5.6 (*n* = 95)	7.7 ± 3.5 (*n* = 22)	7.7 ± 3.2 (*n* = 29)
Lymphocytes (1.0–3.4 nL)	1.4 ± 1.3 (*n* = 79)	1.6 ± 0.9 (*n* = 22)	1.2 ± 1.0 (*n* = 29)
			
**PE on CT**	13.7% (13/95)	13.6% (3/22)	20.7% (6/29)

**Table 3:** Results from Co-RADS analysis.

	All Patients (*n* = 95)		Group A (*n* = 22)		Group B (*n* = 29)	
	Reader 1	Reader 2	Reader 1	Reader 2	Reader 1	Reader 2
**Co-RADS 1**	28	24	0	0	0	0
**Co-RADS 2**	38	29	4	1	6	1
**Co-RADS 3**	9	17	1	3	3	3
**Co-RADS 4**	7	12	4	6	7	12
**Co-RADS 5**	6	6	6	5	6	6
**Co-RADS 6**	7	7	7	7	7	7

**Table 4:** Clinical outcome of COVID-19 patients.

	Group A (*n* = 22)			Group B (*n* = 29)		
	No PE (*n* = 19)	PE (*n* = 3)	*p*-Value	No PE (*n* = 23)	PE (*n* = 6)	*p* Value
**Admission to hospital**						
	94.7% (18/19)	100% (3/3)	1.0	91.3% (21/23)	100% (6/6)	1.0
Of which necessitate at least intermediate care	5.2% (1/19)	0% (0/3)	1.0	13.0% (3/23)	16.7% (1/6)	1.0
Of which necessitate intensive care	5.2% (1/19)	0% (0/3)	1.0	4.3% (1/23)	0% (0/6)	1.0
						
**Oxygen therapy**						
	36.8% (7/19)	66% (2/3)	0.544	43.5% (10/23)	66% (4/6)	0.390
Of which necessitate mechanical ventilation	5.2% (1/19)	0% (0/3)	1.0	4.3% (1/23)	0% (0/6)	1.0
						
Length of stay	10.0 ± 8.1	9 ± 3	0.787	9.5 ± 7.6	5.5 ± 4.3	0.302
Discharged at time of data acquisition	78.9% (15/19)	100% (3/3)		82.6% (19/23)	66% (4/6)	
						
Death	5.2% (1/19)	33% (1/3)	0.26	4.3% (1/23)	33% (2/6)	0.1

## Data Availability

The data presented in this study are available on request from the corresponding author. The data are not publicly available for data protection reasons.
